# Refractory Uveitis in Patient with Castleman Disease Successfully Treated with Tocilizumab

**DOI:** 10.1155/2012/968180

**Published:** 2012-11-06

**Authors:** Toshiyuki Oshitari, Fusae Kajita, Aya Tobe, Makiko Itami, Jiro Yotsukura, Takayuki Baba, Shuichi Yamamoto

**Affiliations:** ^1^Department of Ophthalmology and Visual Science, Chiba University Graduate School of Medicine, Inohana 1-8-1, Chuo-ku, Chiba, Chiba 260-8670, Japan; ^2^Department of Surgical Pathology, Chiba Cancer Center, Nitona-cho 666-2, Chuo-ku, Chiba, Chiba 260-8717, Japan

## Abstract

Although multicentric Castleman disease is a rare but life-threatening disease, eye complications are extremely uncommon. We present a case of refractory uveitis accompanied with Castleman disease successfully treated with tocilizumab. A 58-year-old man with Castleman disease was introduced for refractory uveitis to Chiba University Hospital. Large cells were detected in the anterior chamber and increased vascular permeability of retinal vessels has been found in both eyes. Although the patient was treated with oral and eye drop steroid treatment, the uveitis symptoms had not decreased. The serum levels of CRP and IL-6 were increased. The level of IL-6 concentration in the anterior chamber was the same as the serum level of IL-6. The humanized anti-IL-6 receptor-antibody (tocilizumab) was administrated for the patient because of poor general condition. After tocilizumab treatment, large cells in the anterior chamber were undetectable and vascular permeability was improved in FA. The serum levels of CRP and IL-6 decreased and the general condition improved. The side effect of tocilizumab was not observed during the treatment. Tocilizumab treatment was significantly effective for uveitis accompanied with Castleman disease. Although it is extremely rare, uveitis accompanied with Castleman disease may be one of the hallmarks to consider tocilizumab treatment.

## 1. Introduction

Multicentric Castleman disease (MCD) is widespread lymphadenopathy accompanied by chronic fever, severe fatigue, night sweats, weight loss, and anorexia. MCD is a rare but life-threatening disease, and eye complications are extremely rare [1–3]. We present a patient with MCD with refractory uveitis that was successfully treated with tocilizumab, a humanized anti-interleukin 6 (IL-6) receptor antibody. We evaluated the therapeutic effects of tocilizumab by standard ophthalmological examinations, fluorescein angiography (FA), Goldmann perimetry, and optical coherence tomography (OCT).

## 2. Case Report

**Figure 1:**
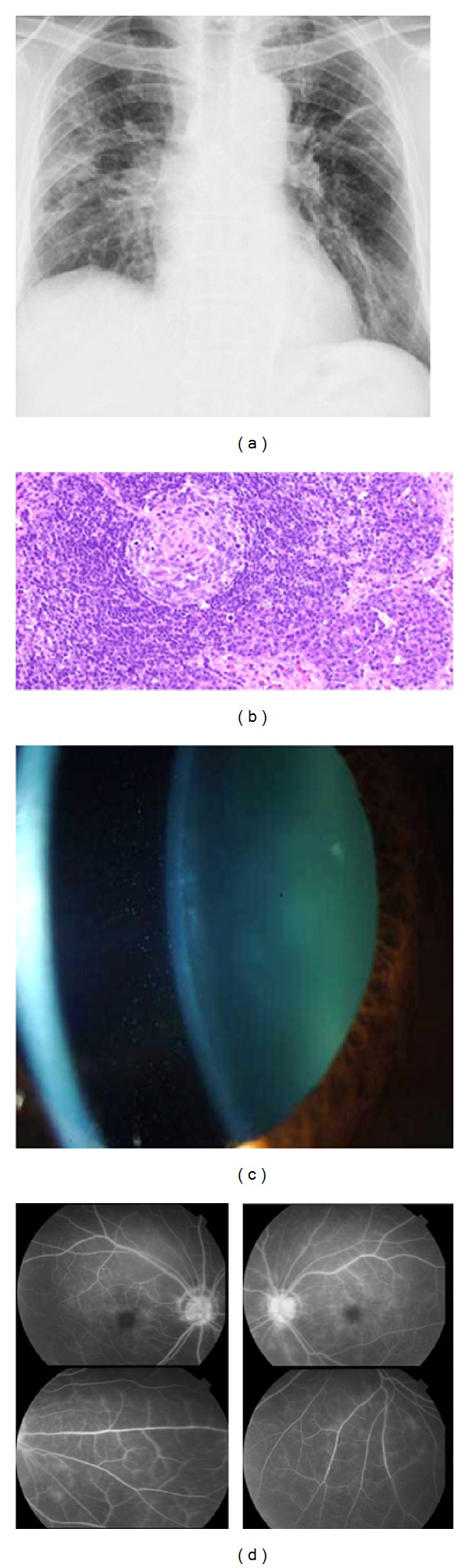
Clinical and pathological findings of a patient with Castleman disease before tocilizumab treatment. Panel (a) shows the thoracic X-ray photograph before tocilizumab treatment. Bilateral hilar lymphadenopathy and infiltrative shadows can be seen in both lungs. The infiltrative shadows remained unchanged for several years. Panel (b) shows the histopathological findings of the pulmonary lymph node performed in the Chiba Cancer Center (×200). Infiltrations of polyclonal plasma cells surrounding the mantle zones and a pattern of concentric deposits with lymphoid cells can be seen. Combined with the typical clinical findings, the patient was diagnosed with Castleman disease. Panel (c) shows a slit-lamp photograph of the anterior chamber before tocilizumab treatment. Large cells (3+) without flare can be seen in the anterior chamber. Panel (d) shows FA findings detecting increased vascular permeability from the retinal vessels and hyper fluorescence of the optic discs in both eyes before treatment.

A 58-year-old man who was diagnosed with MCD in the Chiba Cancer Center was referred to the Chiba University Hospital in December 2010 to treat his refractory uveitis. He had chronic fever, general fatigue, and refractory pulmonary infiltrations (Figure 1(a)) even after oral steroid treatment. The histopathological findings of pulmonary lymph nodes performed in the Chiba Cancer Center showed infiltrations of polyclonal plasma cells surrounding the mantle zones (Figure 1(b)). The level of serum C-reactive protein (CRP) was 21.0 mg/dl, and the level of serum IL-6 was 46.6 pg/ml (normal; <4.0 pg/ml). These clinical and histopathological findings were typical for MCD. His corrected visual acuities were 0.4 (OD) and 0.7 (OS). Large cells without flare were detected in the anterior chamber (Figure 1(c)), and the intraocular pressures (IOPs) were within normal limits. The level of IL-6 in the anterior chamber was 57.0 pg/ml and the level of IL-10 in the anterior chamber was below detectable level. The FA showed leakage from the retinal vessels in both eyes (Figure 1(d)). The OCT findings showed no macular edema in both eyes. These findings were not improved by oral and topical steroid eye drops. The IOPs in the both eyes gradually increased to 34 mmHg (OD) and 36 mmHg (OS) with full medication probably because of steroid response and the entrapment of large cells in the trabecular meshwork.

**Figure 2:**
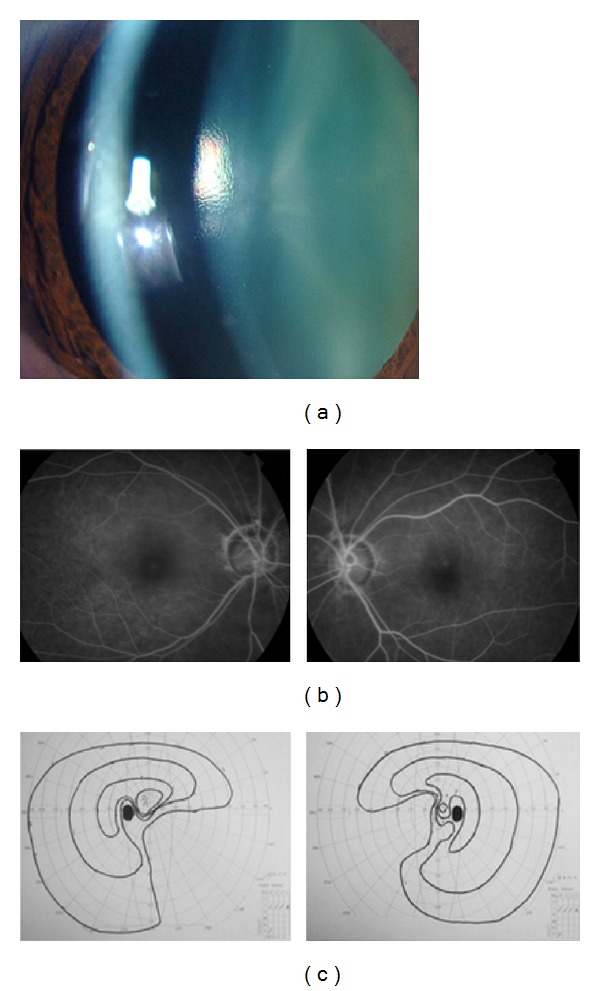
Clinical findings of a patient with Castleman disease after tocilizumab treatment. Panel (a) shows a slit-lamp photograph of the anterior chamber after tocilizumab treatment. Large cells disappeared three months after tocilizumab treatment. Panel (b) shows FA findings after tocilizumab treatment. The degree of leakage improved three months after the tocilizumab treatment. Panel (c) shows nasal visual filed defects observed in Goldmann perimetry in both eyes three months after treatment because of secondary glaucoma before the tocilizumab treatment.

We recommended the patient for tocilizumab because of the uncontrolled IOPs and his poor general conditions. Then, 8 mg/kg per two weeks of tocilizumab was begun in the Chiba Cancer Center. After the tocilizumab treatment, the presence of large cells in the anterior chamber was not detected (Figure 2(a)), and three months after tocilizumab treatment, the vascular leakage of the FA was decreased even without oral and topical steroid treatment (Figure 2(b)). The serum levels of CRP and IL-6 were decreased to 0.0 mg/dl and 11.2 pg/ml, respectively. His general condition was markedly improved. After one year of tocilizumab treatment, no side effects of tocilizumab were observed, and the final visual acuities were 0.5 (OD) and 0.9 (OS). The reduction in the visual acuities was probably because of moderate cataracts. Although the IOPs decreased to within normal limits without antiglaucoma eye drops, nasal visual field defects were detected because of secondary glaucoma (Figure 2(c)). The cup-to-disc ratio was changed 0.7 into 0.9 during the follow-up period. At present, the patient is being continuously treated with 8 mg/kg tocilizumab per 3 weeks to maintain a stable condition.

## 3. Discussion

 Jorge et al. successfully treated the ocular complications of a patient with Castleman disease with immunosuppression and plasmapheresis [4]. However, there is no report demonstrating eye complications of MCD treated with tocilizumab. In general, steroid therapies are ineffective in patients with MCD as in our case. Because the overproduction of IL-6 due to hyperplasia of plasma cells results in the general symptoms of MCD, the anti-IL-6 receptor antibody, tocilizumab, was developed in Japan for the treatment of MCD [5]. Although MCD is rare, such a drug specific for MCD as tocilizumab is very important because MCD is a life-threatening disease. 

 The level of IL-6 in the anterior chamber was almost the same as that in the serum, and after tocilizumab treatment, large cells were undetectable in the anterior chamber. Thus, these large cells might be plasma cells. In this case, tocilizumab treatment was effective for the uveitis accompanying the MCD. Although it is extremely rare, uveitis accompanied with MCD may reflect a generally poor condition of patients with MCD, and ocular complications such as uveitis may be one of the hallmarks to consider tocilizumab treatment.
